# Coexistence of metallic and insulating-like states in graphene

**DOI:** 10.1038/srep08974

**Published:** 2015-03-10

**Authors:** Fang Wu, Jing Huang, Qunxiang Li, Kaiming Deng, Erjun Kan

**Affiliations:** 1 School of Science, Nanjing Forestry University, Nanjing, Jiangsu 210037, P. R. China; 2 Key Laboratory of Soft Chemistry and Functional Materials (Ministry of Education), and Department of Applied Physics, Nanjing University of Science and Technology, Nanjing, Jiangsu 210094, P. R. China; 3 School of Materials and Chemical Engineering, Anhui University of Architecture, Hefei, Anhui 230022, P. R. China; 4 Hefei National Laboratory for Physical Sciences at the Microscale, University of Science and Technology of China, Hefei, Anhui 230026, P. R. China

## Abstract

Since graphene has been taken as the potential host material for next-generation electric devices, coexistence of high carrier mobility and an energy gap has the determining role in its real applications. However, in conventional methods of band-gap engineering, the energy gap and carrier mobility in graphene are seemed to be the two terminals of a seesaw, which limit its rapid development in electronic devices. Here we demonstrated the realization of insulating-like state in graphene without breaking Dirac cone. Using first-principles calculations, we found that ferroelectric substrate not only well reserves the Dirac fermions, but also induces pseudo-gap states in graphene. Calculated transport results clearly revealed that electrons cannot move along the ferroelectric direction. Thus, our work established a new concept of opening an energy gap in graphene without reducing the high mobility of carriers, which is a step towards manufacturing graphene-based devices.

Two-dimensional atomic materials, which are consisted of one or several atomic layers, have attracted great research interests in recent years^1^,^2^,^3^. Since graphene has great potential in electric, optical, mechanical, and catalytic applications^4^,^5^,^6^, it has been investigated from atomic growth^7^,^8^,^9^ to the electronic manipulation^10^,^11^,^12^,^13^. In particularly, the massless fermions in graphene have high mobility up to 2.5 × 10^5^ cm^2^V^−1^s^−1^, which exceed that of all conventional semiconductors used in today's devices. This fascinating character of extremely high carrier mobility endows graphene great advantage for electronic applications.

As we know, in single layer graphene, valence and conduction bands, which are dominated by the nearest-neighbor carbon *p_z_*-*p_z_* bonding (Fig. 1a), linearly intersected at the Dirac points (K) in reciprocal space. Consequently, valence and conduction bands are degenerate at Dirac point, namely, graphene has an intrinsic property of semimetal. To switch off the conducting channels of graphene in transistors or other similar devices, huge efforts have been spent to pursue semiconducting graphene^14^,^15^,^16^,^17^,^18^,^19^,^20^,^21^. From the viewpoint of geometric structures, current efforts can be divided into two parts: geometric modifications (Fig. 1b) and electronic manipulation (Fig. 1c). For both methods of band gap opening, the carrier mobility is inevitably reduced, and it is difficult to control the magnitude of band gap through such methods.

It is quite troublesome that opening of the bandgap and the mobility of carriers in graphene are seemed to be the two terminals of a seesaw, which are difficult to coexist with each other. However, one of the ultimate objectives of bandgap engineering in graphene is the realization of stopping the flow of electrons through graphene without breaking the character of high mobility. Naturally, it is highly desired to introduce new concepts or ideas to engineer the bandgap of graphene.

In this study, based on first-principles calculations, we established a new route to opening the bandgap in graphene. Taking ferroelectric OH-BNSL monolayer as the substrate of graphene, we found that the linear dispersion of energy bands near the Dirac points is well preserved, demonstrating the existence of Dirac fermions in graphene. Importantly, the ferroelectric OH-BNSL layer periodically modulates the charge density distribution of graphene, and leads to a pseudogap in graphene. The calculated transport properties clearly revealed that the flow of electrons in graphene along the orientation of hydroxyls on BNSL is almost prohibited within an energy range, displaying an insulating-like character. While in the perpendicular direction, the carriers can freely transport as that in intact graphene. Thus, our results explored the amazing coexistence of metallic and insulating-like states in graphene without reducing the high mobility of carriers, and will benefit today's development in graphene-based electric devices.

To make graphene/ferroelectrics bilayer, it is necessary to prepare lattice-matched ferroelectric materials. It is noted that many works have reported that chemical functionality is an effective method tune the physical and chemical properties of graphene and h-BN. Recently, Sainsbury et al. took a two-step procedure to chemically functionalize hexagonal boron nitride single layer (BNSL)^22^. Using organic peroxide as the source of radical species, they found that alkoxy groups are initially grafted to boron atoms, and the subsequent hydrolytic defunctionlization of the groups yield hydroxyl-functionalized BNSL (OH-BNSL), and the chemical groups are homogeneously distributed on the surface of BNSL. As revealed in our previous works, hydroxyl-functionalized graphene is ferroelectric material^23^. Thus, as expected, OH-BNSL should be a ferroelectrics.

For the experimentally synthesized hydroxyl-functionalized BNSL (OH-BNSL), hydroxyl groups are connected with boron atoms. However, the orientation of hydroxyl groups on BNSL is not explored. As shown in Fig. 2, we used a supercell to examine three possible arrangements of hydroxyl groups on BN layer. Through ion relaxations and total energy calculations, we found that the motif of all hydroxyl groups pointing in the same direction (Fig. 2c) has the lowest energy, and the structures shown in Fig. 2a and Fig. 2b are higher in energy by 36 and 70 meV, respectively. Since each hydroxyl group has an intrinsic electric dipole, a spontaneous electric polarization is naturally expected when such hydroxyl groups are homogeneously distributed on the surface of BNSL and point in the same direction. The further calculations show that the electric polarization of OH-BNSL is about 4.68 × 10^−7^ *μ*C/cm.

Now we turn to explore the electronic properties of graphene/OH-BNSL bilayer, in which graphene is covered on the surface of ferroelectric OH-BNSL. As shown in Fig. 3a, two possible structures are adopted as the initial configurations. The total energy calculations show that the structure with carbon atoms locating above boron atoms (labeled as “B”) is slightly stable than that of carbons atoms above nitrogen atoms ((labeled as “N”), the energy difference is about 4 mV per hydroxyl group). Besides, compared with that of graphene/BN bilayer (around 3.4 Å), the inter-layer distance of graphene/OH-BNSL is much larger due to the existence of hydroxyl group. The distance between graphene and OH-BNSL sheets is predicted to be about 5.5 Å. Thus, it is not expected that there is a chemical interaction between graphene and OH-BNSL layers.

As stated in our previous reports^19^, charge transfer between graphene and substrates plays an important role to open an energy gap in graphene. However, for graphene-OH-BNSL bilayer, inter-layer charge transfer is neglectable. Consequently, the energy gap cannot be opened in graphene. Such picture is well confirmed by our DFT calculations. As shown in Fig. 3b, the plotted band dispersion of graphene/OH-BNSL bilayers clearly demonstrated that valence and conduction bands linearly intersected around Dirac point (K), and degenerate in energy at Dirac point. As a consequence of the linear band behaviors at Dirac point, graphene is still a semi-metal with high mobility of carriers. To confirm the high mobility, we calculated the Fermi velocity, which is very close to that of free-standing graphene (around 10^6^ m/s).

Although the character of linear band dispersion and zero-energy gap in graphene is not disturbed by the OH-BNSL, it is cursory to deduce that OH-BNSL has little effect on the electronic property of graphene. Since OH-BNSL introduces a periodic electric potential along the ferroelectric direction (the orientation of hydroxyls) in graphene, the charge density of graphene should be modulated by this periodic electric potential. As shown in Fig. 4a, the plotted charge density of both conductive band minimum (CBM) and valence band maximum (VBM) around the Fermi level shows chain-like patterns, which are perpendicular to the ferroelectric direction. To show how the charge chains are separated, we used a one-dimensional charge density ρ(y), which is defined as 
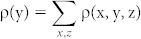
, where y means the direction of ferroelectric polarization, x means the direction perpendicular to y, and z means the direction perpendicular to xy plane. As plotted in Fig. 4b, between the charge chains, both the CBM and VBM have much less charge density. Especially for CBM, our calculated results explored that the charge density between chains is zero.

Generally, the carriers transport will be modified by the charge density wave. As we know, along the direction of chain-like charge patterns, the carriers can much easier be transported because of the strong overlapping of nearest-neighbor wave function. Besides, the calculated band dispersion has demonstrated the existence of Dirac fermions, namely, such carriers have a high mobility. While for the direction perpendicular to the chain-like charge patterns, carriers are difficult to be transferred from one chain to another one because of the weaker overlapping of nearest-neighbor wave function, namely, there is a “pseudogap” along this direction although there is no real energy gap in the electronic structures.

To verify the above predictions, we perform first-principles calculations combined with non-equilibrium Green's function method to explore the transport properties of two graphene/OH-BNSL bilayer junctions, as shown in inserts of Fig. 4, in which hydroxyl groups parallel to or vertically pointing in transport direction, and labeled with **PJ** and **VJ**, respectively. The calculated zero-bias transmission functions of two proposed graphene/OH-BNSL bilayer junctions are plotted in Fig. 4. It is clear that the transmission coefficents through **VJ** are significant larger than that of **PJ** within the energy range from −0.25 to 0.12 eV. For example, at the Fermi level, the transmission coefficient of **VJ** is about 1.6, and the corresponding value of **PJ** is very small (less than 10^−4^), which stands for the metallic and insulating states, respectively. These results show that the transport direction of graphene/OH-BNSL bilayer system depends on the orientation of hydroxyl groups on BNSL. The metallic channel coexists with an insulating-like one in graphene/OH-BNSL bilayer system, which well confirms our above electronic structure calculations. Moreover, the metallic state can be switched into the insulating-like state by changing the orientation of hydroxyl groups on BNSL.

It should be noted that the “pseudogap” explored in graphene/OH-BNSL bilayer is totally different with that of previous reports^18^,^19^,^20^,^21^,^22^. First of all, in this study, the “pseudogap” does not mean a real energy gap in the electronic structures. The prohibited transport of carriers originates from the charge screening. Secondly, it is the first time to report the coexistence of metallic and insulating-like states in the graphene. Conventionally, the electronic structure of graphene is only can be tuned from metallic state to insulating one, or vice versa. Thirdly, the mechanics response for the “pseudogap” is quite different with the previous results^18^,^19^,^20^,^21^,^22^. As shown in Fig. 1, an energy gap in graphene is opened by the breaking the degenerate 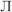 and 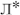
* orbitals in conventional methods. While in the present investigation, the “pseudogap” is produced by the anisotropic charge distribution, induced by the ferroelectric substrate.

Since the electronic properties are dominated by the carriers around Fermi level, and it is inevitable that graphene is doped with electrons, it is necessary to check whether such “pseudogap” state is well reserved. As explored in previous reports, experiments have achieved doping concentration at 10^13^/cm^2^. Therefore, in the following studies, we will limit the electron concentration below 1.0 × 10^13^/cm^2^. As shown in Fig. 5a, linearly intersected bands are well preserved under three kinds of electron doping (0.2 × 10^13^/cm^2^, 0.5 × 10^13^/cm^2^, 1.0 × 10^13^/cm^2^). Importantly, in all cases, the charge density around Fermi level has the chain-like patterns (Fig. 5b). To get more details of the charge distribution, in Fig. 5c, we plotted the one-dimensional charge density ρ(y), which is the same picture as discussed in Fig. 4b. It is quite clear that the charge density is almost zero between two charge chains. Consequently, the electrons are not allowed to move along this direction, and “pseudogap” state is well kept under carriers doping.

In summary, based on first-principles calculations, we found that the electric polarization of functional boron nitride tunes the electronic structure of graphene in graphene/OH-BNSL bilayer without destroying the Dirac cone, making the electronic structure of graphene anisotropic. The calculated transport results clearly explored the existence of an insulating-like state in this functionalized graphene. Thus, our work illustrates a new concept of opening an energy gap in graphene, which is a step towards manufacturing graphene-based devices.

## Methods

Our first-principles calculations were based on spin-polarized density functional theory (DFT) using the generalized gradient approximation (GGA) known as PW91^24^, implemented in the Vienna *ab initio* simulation package (VASP) code. The projected augmented wave (PAW) method^25^,^26^ with a plane-wave basis set was used. We applied periodic boundary conditions and a vacuum space of about 20 Å along the z direction in order to avoid the artificial interaction between two layers in nearest-neighbor unit cells. All of the structures were relaxed using the conjugated gradient method without any symmetric constraints. To consider the possible anti-parallel model of hydroxyl groups, all calculations were performed by 4 × 4 × 1 supercell of graphene. The Monkhorst-Pack special k-point meshes of 7 × 7 × 1 was used for the supercell to represent the reciprocal spaces. We set the energy cutoff and convergence criteria for energy and force to be 500 eV, 10^−4^ eV, and 0.01 eV/Å, respectively. The accuracy of our simulation was tested through a comparison of the presented results with previous theoretical results.

The transport calculations for graphene/OH-BNSL bilayer were carried out by using the fully self-consistent non-equilibrium Green's function method combined with first-principles calculations, implemented in ATK package^27^,^28^. We calculated the zero-bias transmission function, T(E,V), through the junctions. Here, T(E, V) is defined as Tr[Γ_L_(E,V)G(E,V)Γ_R_(E,V)G^+^(E,V)], in which Γ_L(R)_ stands for the coupling matrix between the left(right) electrode and the central scattering region, and G(E,V) is the retarded Green's function of the scattering region. In our calculations, we adopted Troullier-Martins nonlocal pseudopotential and double-zeta plus polarization basis sets for all elements in the junctions. The grid integration was defined by an energy cutoff of 150 Ry, while the exchange and correlation functional was treated at the level of Ceperley-Alder local-density approximation. Test calculations with more k-points and larger cutoff energy yield the similar results.

## Author Contributions

E.K. conceived the project. F.W. performed the calculations of electronic properties. J.H. studied the electric conductivity and calculated the I–V curve. F.W., J.H., Q.L., K.D. and E.K. co-wrote the paper, and all authors contributed to the discussion and preparation of the manuscript.

## Figures and Tables

**Figure 1:**
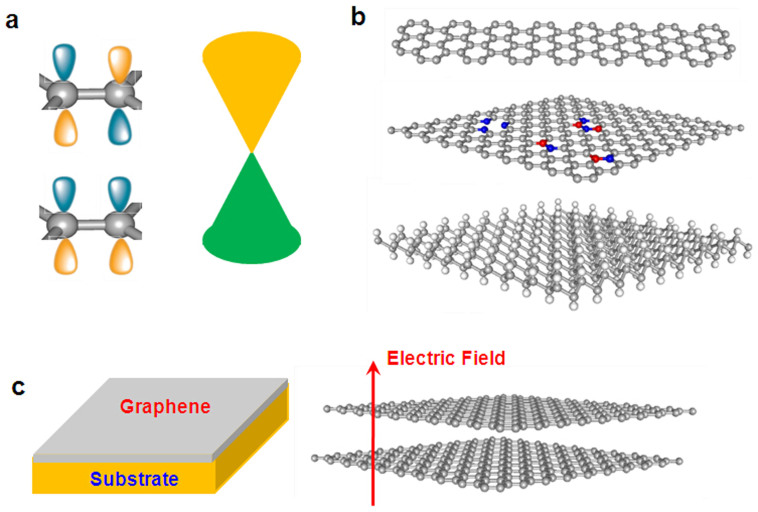
Energy gap of graphene. (a) The schematic diagram of band dispersion at Dirac points (K) and the orbitals contributing to the conductive band minimum and valence band maximum. (b) Bandgap engineering through geometric modifications, the top one is graphene nanoribbons, the middle one is doped graphene (red and blue balls represent doped atoms), and the bottom one is the hydrogenated graphene. (c) Bandgap engineering through electronic manipulations, the left one means heterostructures formed by graphene and substrate, and the right one is graphene bilayer under an external electric field.

**Figure 2:**
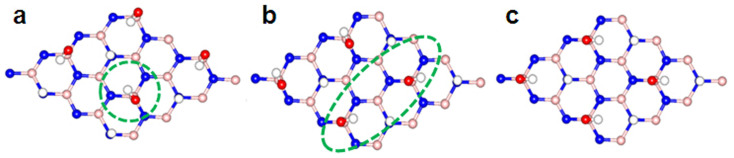
Three possible arrangements of hydroxyl groups in hydroxyl-functionalized BNSL. (a) One of four hydroxyl groups points in different direction (indicated by the green cycle), (b) Two of four hydroxyl groups point different direction, (c) all hydroxyl groups point the same direction. Blue, orange, red and white balls are nitrogen, boron, oxygen, and hydrogen atoms, respectively.

**Figure 3:**
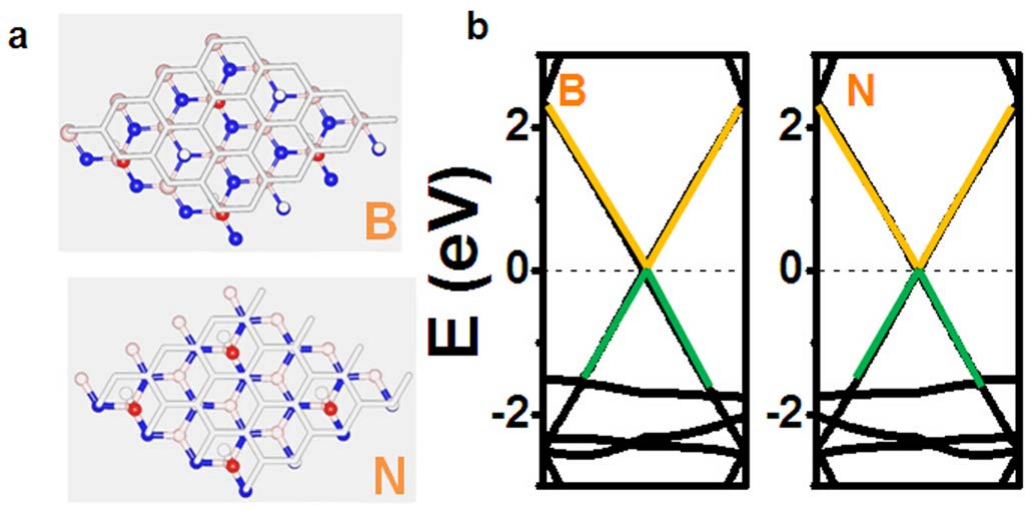
Electronic properties of graphene/OH-BNSL bilayer.(a) Two possible structures are adopted in our studies, label “B” means the structure of carbon atoms locating above boron atoms, and label “N” means that of carbon atoms locating above nitrogen atoms. Blue, orange, red and white balls are nitrogen, boron, oxygen, and hydrogen atoms, respectively. (b) The calculated band dispersion for “B” and “N” structures.

**Figure 4:**
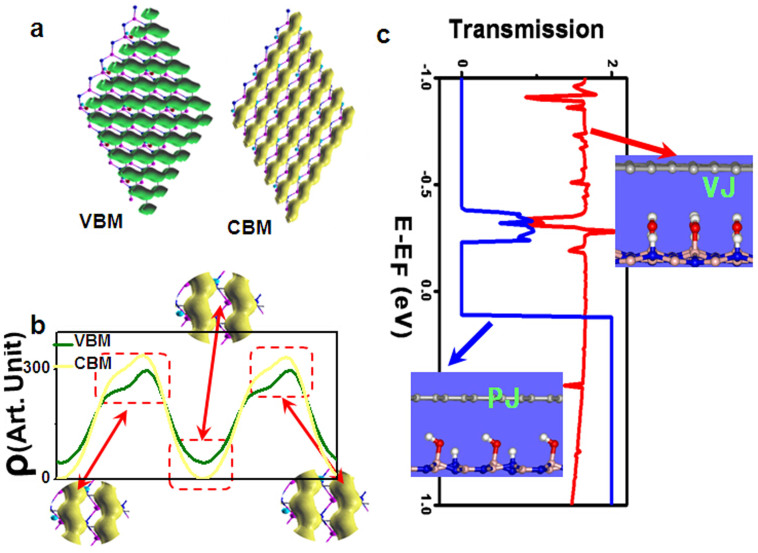
(a) The plotted the charge density of conductive band minimum (CBM) (right) and valence band maximum (left) at Dirac point (K). In both cases, the charge density is plotted with isovalue of 1.5 × 10 ^− 4^ e/Å^3^. (b) The plotted one-dimensional charge density distribution ρ (y), which is defined as ρ(y) = 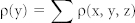
ρ(x, y, z), where y means the direction of ferroelectric polarization, x means the direction perpendicular to y, and z means the direction perpendicular to xy plane. (c) Calculated zero-bias transmission function of graphene/OH-BNSL bilayer along two different directions. The right and left inserts, labelled with VJ and PJ, stands for two molecular junctions, in which the hydroxyl groups parallel to or vertically pointing in transport direction, respectively.

**Figure 5:**
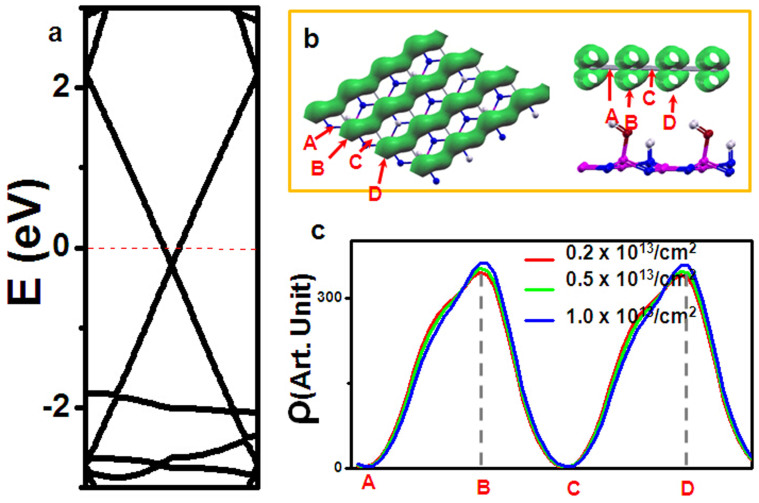
(a) The calculated band dispersion of graphene/OH-BNSL bilayer with carrier concentration of 0.5 × 10^13^/cm^2^. (b) The plotted the charge density of conductive band below Fermi level, and the charge density is plotted with isovalue of 1.5 × 10 ^− 4^ e/Å^3^. (c) The plotted one-dimensional charge density distribution ρ (y), which is defined as ρ(y) = 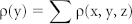
ρ(x, y, z), where y means the direction of ferroelectric polarization, x means the direction perpendicular to y, and z means the direction perpendicular to xy plane. Along the direction, A, B, C, D have been indicated by the arrows in Fig. 5b.
